# Compound heterozygous *PLA2G6* loss-of-function variants in Swaledale sheep with neuroaxonal dystrophy

**DOI:** 10.1007/s00438-020-01742-1

**Published:** 2020-11-06

**Authors:** Anna Letko, Ben Strugnell, Irene M. Häfliger, Julia M. Paris, Katie Waine, Cord Drögemüller, Sandra Scholes

**Affiliations:** 1 Institute of Genetics, Vetsuisse Faculty, University of Bern, 3001 Bern, Switzerland; 2 Farm Post Mortems Ltd, Hamsterley House, Hamsterley, Bishop Auckland, Durham DL13 3QF UK; 3 University of Nottingham, College Road, Sutton Bonington, Loughborough, LE12 5RD UK; 4 SRUC Consulting Veterinary Services, Pentlands Science Park, Bush Estate Loan, Penicuik, Midlothian EH26 0PZ UK

**Keywords:** *Ovis aries*, Neurogenetic disorder, Rare disease, Compound heterozygosity, Precision medicine

## Abstract

Sporadic occurrences of neurodegenerative disorders including neuroaxonal dystrophy (NAD) have been previously reported in sheep. However, so far no causative genetic variant has been found for ovine NAD. The aim of this study was to characterize the phenotype and the genetic aetiology of an early-onset neurodegenerative disorder observed in several lambs of purebred Swaledale sheep, a native English breed. Affected lambs showed progressive ataxia and stiff gait and subsequent histopathological analysis revealed the widespread presence of axonal spheroid indicating neuronal degeneration. Thus, the observed clinical phenotype could be explained by a novel form of NAD. After SNP genotyping and subsequent linkage mapping within a paternal half-sib pedigree with a total of five NAD-affected lambs, we identified two loss-of-function variants by whole-genome sequencing in the ovine PLA2G6 gene situated in a NAD-linked genome region on chromosome 3. All cases were carriers of a compound heterozygous splice site variant in intron 2 and a nonsense variant in exon 8. Herein we present evidence for the occurrence of a familial novel form of recessively inherited NAD in sheep due to allelic heterogeneity at *PLA2G6.* This study reports two pathogenic variants in *PLA2G6* causing a novel form of NAD in Swaledale sheep which enables selection against this fatal disorder.

**Electronic supplementary material:**

The online version of this article (10.1007/s00438-020-01742-1) contains supplementary material, which is available to authorized users.

## Introduction

Neuroaxonal dystrophy (NAD) comprises a clinically and genetically heterogeneous group of neurodegenerative diseases of central nervous system (Hayflick et al. 2018). It is characterized by progressive signs of neurological dysfunction including ataxia, hypermetria, proprioceptive deficits, head incoordination and tremors. The characteristic neuropathological changes involve formation of axonal swellings (spheroids) in specific regions, usually relay nuclei, of the brainstem and spinal cord (Sisó et al. 2006). In humans, this condition is usually described as neurodegeneration with brain iron accumulation (NBIA) or infantile neuroaxonal dystrophy (INAD; OMIM PS234200) and is most frequently monogenic recessively inherited. The genetic basis is described for ~ 85% of patients diagnosed with NBIA and involves eight genes (Hayflick et al. 2018). Human NBIA2A (INAD1) and NBIA2B are associated with autosomal recessive variants in the *PLA2G6* gene (OMIM 256600). Phospholipase A2 group VI gene (*PLA2G6*) encodes 85/88 kDa calcium-independent phospholipase A2, an enzyme that catalyzes the hydrolysis of phospholipids to produce free fatty acids, and has a critical role in cell membrane homeostasis (Baburina and Jackowski 1999).

In veterinary medicine, sporadic cases of NAD have been previously described in sheep (OMIA 000715-9940), as well as cattle (Hanshaw et al. 2015), dogs (OMIA 000715-9615), cats (OMIA 000715-9685), rabbits (OMIA 000715-9986), and horses (OMIA 000715-9796). Although, there is a variation in the age of affected lambs in the previous reports of ovine NAD cases, the clinicopathological features are comparable in all the different sheep breeds including Merino (Harper and Morton 1991; Kessell et al. 2012), Suffolk (Cordy et al. 1967), Romney (Nuttall 1988), Perendale (Nuttall 1988), and Coopworth (Nuttall 1988), as well as multiple crossbred Merino-Border Leicester × Polled Dorset lambs (Finnie et al. 2014; Hawes et al. 2017). However, the underlying genetic variants to date have been characterized only in dogs, representing four breed-specific autosomal recessive variants in *MFN2* (Fyfe et al. 2011), *TECPR2* (Hahn et al. 2015), *PLA2G6* (Tsuboi et al. 2017), and *VPS11* (Lucot et al. 2018) genes.

The aim of this study was to characterize the phenotype and the genetic aetiology of an early-onset neurodegenerative disorder observed in several lambs of purebred Swaledale sheep, a native English breed. Herein we present evidence for the occurrence of a familial novel form of recessively inherited NAD due to two compound heterozygous loss-of-function variants in ovine *PLA2G6*, which enables selection against this fatal disorder.

## Methods

### Animals

This study did not require official or institutional ethical approval as it was not experimental. A total of 71 purebred Swaledale sheep from one flock were used in this study, including 52 ewes, 1 ram, 5 NAD-affected lambs, and 13 apparently normal offspring. All lambs were sired by the same ram and also shared the same maternal grandsire. Blood samples were taken from all available animals. Additionally, tissue samples (brain and spinal cord) were collected postmortem from 2 representative cases for further analyses. Genomic DNA was isolated from blood using the Maxwell RSC whole blood DNA kit (Promega).

### Neuropathology

Samples of brain and spinal cord from affected lambs were fixed in 10% neutral-buffered formalin for histopathological analysis. Representative samples of brain including cerebral cortex (frontal, parietal, temporal and occipital lobes), striatum, hippocampus, rostral and caudal thalamus, rostral and caudal midbrain, cerebellum (vermis and hemisphere), rostral and caudal medulla and spinal cord including cervical segments 3 and 7, thoracic segment 7 and lumbosacral intumescence were routinely processed to 5 µm haematoxylin-and-eosin (HE)-stained sections for histopathological examination. Selected affected regions were immunolabelled for amyloid precursor protein (APP) as described previously (Garcia et al. 2015).

### SNP array genotyping and analyses

Genomic DNA of 14 sheep (5 ewes, 1 ram, 5 NAD-affected, and 3 unaffected lambs) was genotyped on the Illumina OvineSNP50 BeadChip array. All genome positions refer to the ovine reference genome assembly Oar_rambouillet_v1.0. PLINK v1.9 software (Chang et al. 2015) was used for quality control of the genotyping data, parentage confirmation, as well as autozygosity mapping. All samples had good quality as indicated by > 90% call rate per individual. The final dataset included 44 066 SNP markers after pruning based on missing genotype calls per marker (> 10%). Non-parametric linkage analysis was carried out using the Merlin software (Abecasis et al. 2002) to test for co-segregation of any chromosomal regions and the NAD phenotype.

### Whole-genome sequencing and variant calling

Whole-genome sequencing data of two parent–offspring trios (the sire, two dams, two NAD-affected lambs) were obtained after the preparation of a PCR-free fragment library at an average 21.5 × coverage. Fastq-files were mapped to the ovine reference genome assembly Oar_rambouillet_v1.0. Variant calling of single nucleotide and small indel variants was performed using NCBI annotation release 103 to predict their functional effects as described before (Paris et al. 2019). Private protein-changing variants present in the two NAD-affected animals were identified by comparison with 60 publically available control genomes, designated as local control cohort, which were produced during other ongoing projects of our group (Online Resource 3). In addition, the Sheep genomes project variant database of further 453 samples (Naval-Sanchez et al. 2018) available from the European Nucleotide Archive (ENA), herein designated as a global control cohort, was searched for the presence of identified variants. The Integrative Genome Viewer (Thorvaldsdóttir et al. 2013) was used for confirmation of the identified sequence variants and for visual inspection to exclude any structural variants in the critical region.

### RNA isolation and RT-PCR

RNA was extracted from samples of the spinal cord, sciatic nerve, and cerebellum of three NAD-affected lambs using the RNeasy Fibrous Tissue Mini Kit (Qiagen). The tissue was first finely crushed by mechanically using TissueLyser (Qiagen), and RNA was extracted by centrifugation following the instructions of the manufacturer. Total mRNA was reverse-transcribed into cDNA using the SuperScript IV Reverse Transcriptase kit (Thermo Fisher Scientific) with oligo d(T) primers. RT-PCR was carried out using primers spanning the different exons' boundaries (Additional File 5) of the *PLA2G6* gene, and the sequences of the RT-PCR products were obtained by Sanger sequencing as described below. *ACTB* was included as a reference gene control (Additional File 5).

### PCR and targeted genotyping

Sanger sequencing was used to confirm the WGS results and to perform targeted genotyping for the identified *PLA2G6* variants as well as to sequence the obtained RT-PCR products. Primers were designed using the Primer-BLAST tool (Ye et al. 2012). After amplification with AmpliTaqGold360Mastermix (Thermo Fisher Scientific), the purified PCR products were directly sequenced on an ABI3730 capillary sequencer (Thermo Fisher Scientific). The sequence data were analyzed using Sequencher 5.1 software (GeneCodes). All primer sequences are available in Additional File 5.

## Results

### Phenotype

In May 2017, five 6-week-old purebred Swaledale lambs presented with ataxia and a stiff gait, which over a period of days progressed to tremor, lateral recumbency, paddling and nystagmus (Additional File 1). The parents of all available affected animals were healthy. The lambs were part of a 300-ewe Swaledale flock of which every year 60 Swaledale shearlings (18 month old females) were all bred to one Swaledale ram, and all older sheep were bred to a Bluefaced Leicester ram to produce North Country Mules for sale as breeding sheep. The Swaledale ram was changed every 2 years so that he did not mate his own daughters. Thus, all Swaledale shearlings were also sired by the same ram (the previous one, which was the maternal grandsire of the affected lambs). Clinical signs were confined to the purebred Swaledale lambs, in which group an estimated 15 NAD-affected lambs were seen from a total of 110 lambs. Some lambs suspected to have been affected were found dead having fallen into a ditch. Lambs of both genders were affected. No clinical signs were seen at any time in crossbred lambs. After mating (in November 2016), all sheep were managed as one group until lambing, which occurred outside in April 2017. Border disease virus was not detected by PCR in any of the affected lambs.

### Neuropathology

**Fig. 1:**
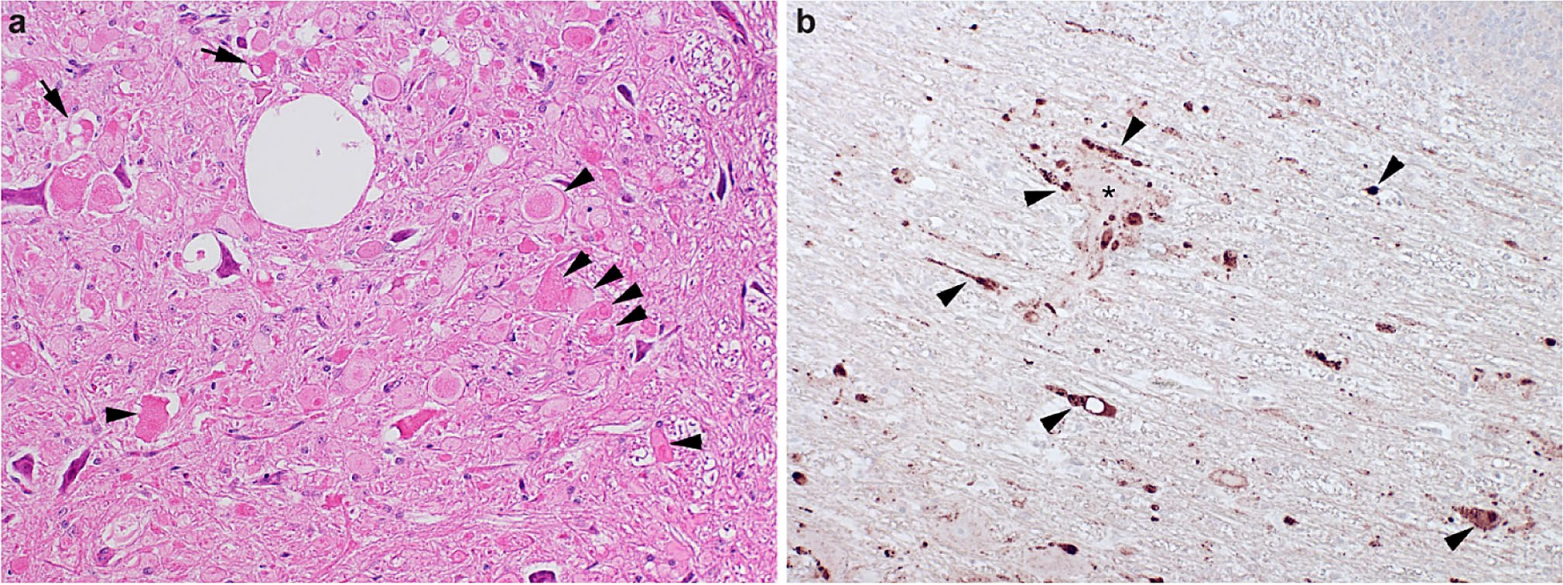
Neuropathology of neuroaxonal dystrophy in a 6-week-old Swaledale lamb. **a** Numerous, often contiguous, swollen nerve fibers (spheroids, arrowheads), some of which are vacuolated indicate a finding typical of neuroaxonal dystrophy in the accessory cuneate nucleus (HE staining). **b** Immunohistochemical labelling for amyloid precursor protein (APP), the expression of which is upregulated in injured axons, demonstrates numerous swollen nerve fibres (arrowheads), some surrounding nerve cell bodies (asterisk), in the cerebellar roof nuclei (APP immunohistochemistry)

Neuropathological analysis detected widespread spheroid formation predominantly involving brainstem grey matter, including accessory cuneate, olives, rostral colliculus, lateral geniculate body, caudal colliculus, medial geniculate body, and oculomotor nuclei (Fig. 1a). Spheroid formation was also present in the cerebellar nuclei, cortex with proximal Purkinje axonal spheroid (torpedo) formation and variable smaller spheroid formation in the internal granule cell layer and Purkinje neuronal dendrites, accompanied by occasional Purkinje neuronal degeneration and loss with focal gliosis in the Bergmann layer. Spheroid formation was also detectable to a much lower extent in the white matter associated with grey matter lesions. In the spinal cord, spheroid formation was most prominent in the intermediate grey matter at segment T7. Immunohistochemistry for amyloid precursor protein, a known marker of axonal injury (Garcia et al. 2015), highlighted the presence of widespread nerve fiber swelling including perineuronal sites (Fig. 1b). Thus, the observed clinicopathological phenotype could be explained by a novel form of neuroaxonal dystrophy (NAD).

### SNP data analyses

**Fig. 2:**
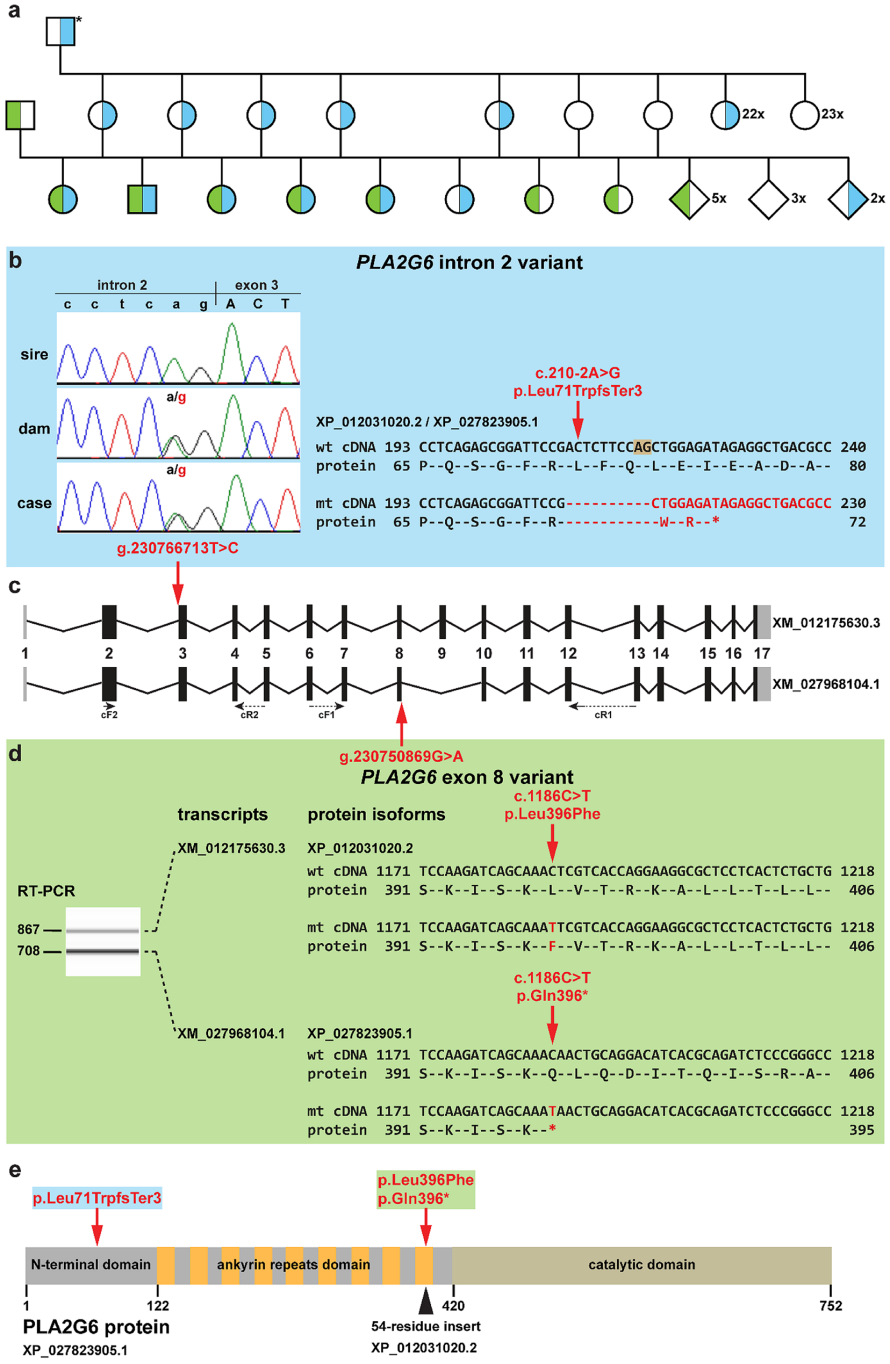
Experimental confirmation of the *PLA2G6* variants in a pedigree of Swaledale sheep. **a** Pedigree showing the NAD-affected lambs as symbols with filled shapes of green and blue. Healthy animals carrying the *PLA2G6* intron 2 variant (c.210-2A > G) are shown as blue half-filled symbols. Carriers of the *PLA2G6* exon 8 variant (c.1186C > T) are shown as green half-filled symbols. Animals genotyped as non-carriers for both variants are indicated as open symbols. The genotype of the maternal grandsire (*) was deduced based on the offspring. **b** Features of the *PLA2G6* intron 2 variant. Note that the electropherograms presented on the left show that the mutant allele is present in heterozygous form in the affected offspring and its mother and absent in the father. The variant affects the conserved acceptor splice site at the end of intron 2 which activates a cryptic splice site at the beginning of exon 3 (shown in orange) that leads to a frameshift and a premature stop codon. **c** Schematic representation of the two different ovine *PLA2G6* transcripts. The four primers used for RT-PCR are indicated with arrows. **d** Features of the *PLA2G6* exon 8 variant. RT-PCR products obtained from cerebellum of an NAD-affected animal. The two bands correspond to the two *PLA2G6* transcripts and the predicted effects of the variant on the two encoded protein isoforms (missense, nonsense) are shown on the right. **e** The schematic representation of the ovine PLA2G6 protein adapted from (Malley et al. 2018). The longer isoform contains a proline-rich insert at the end of the ankyrin repeat domain. Note that the intron 2 variant truncate significant parts of both isoforms. The exon 8 variant leads to the lack of the C-terminus of the short isoform only, whereas it affects only a single amino acid of the longer PLA2G6 protein

Array genotypes of 50 k single-nucleotide polymorphism (SNP) markers were available for 14 Swaledale sheep including 5 NAD-affected and 3 unaffected lambs, 5 dams and their assumed sire. The sampled ram was confirmed as a sire of all eight lambs. Maternity status was investigated for the five selected ewes to determine the potential dams of the NAD-affected lambs. This enabled the construction of a pedigree (Fig. 2a) and a selection of five animals forming two complete parent–offspring trios for whole-genome sequencing (WGS). Furthermore, SNP genotyping data confirmed the reported common origin of the ewes sired by a single ram, which was used in the flock in the previous years. Based on the pedigree analysis, we assumed recessive inheritance and carried out autozygosity mapping. However, this analysis revealed no intervals of extended homozygous regions with alleles shared by all five NAD-affected animals. In a further attempt to map the disease-associated locus, a non-parametric linkage analysis was performed, which resulted in 41 NAD-linked genome regions showing positive LOD scores (Online Resource 2) in the studied Swaledale pedigree.

### Whole-genome sequence analysis

We obtained whole-genome sequences of two NAD-affected lambs, their two dams, and the sire. We hypothesized that a rare breed-specific deleterious variant is responsible for the described NAD phenotype. Therefore, filtering for protein-changing variants present only in these five Swaledale sheep compared to a local control cohort of 60 available genomes from 16 unrelated breeds (Online Resource 3) was performed. Thereby six variants inherited in a simple recessive way or as compound heterozygous remained (Online Resource 4). Additional filtering against a global control cohort of 453 genomes of 54 other sheep breeds (Naval-Sanchez et al. 2018) resulted in only four variants left (Online Resource 4). Among these four, only one variant on chromosome 15 (g.50640254C > T) was homozygous in both affected offspring and heterozygous in all sequenced parents fitting to simple recessive inheritance. As this variant is neither located in a NAD-linked genome region nor a shared homozygosity segment was identified before, we considered this as a less likely candidate, in addition to the fact that the affected *LOC101121706* is an uncharacterized gene. Another missense variant in the *HERC1* gene, known to be associated with macrocephaly, dysmorphic facies, and psychomotor retardation in humans (OMIM 605109), was found heterozygous in both cases and their sire, whereas the second missense variant in the *HERC1* gene appeared also in the global control cohort. We finally excluded these two variants based on the absence of detected genetic linkage to the genome region on chromosome 7 (Online Resource 2). The two other remaining private variants were both located on chromosome 3 (g.230766713T > C and g.230750869G > A) and were predicted as loss-of-function variants of the *PLA2G6* gene (Fig. 2). Variants in *PLA2G6* are known to be associated with autosomal recessive early-onset forms of neurodegeneration, such as infantile neuroaxonal dystrophy 1 or Parkinson disease 14 (OMIM 603604). Both *PLA2G6* variants were heterozygous in the two sequenced NAD-affected offspring, while the two dams were heterozygous for only the variant in *PLA2G6* intron 2 (c.336-2A > G) and the sire was heterozygous for the variant in *PLA2G6* exon 8 (c.1312C > T; Fig. 2a). Additionally, these two *PLA2G6* variants map to one of the identified NAD-linked regions on chromosome 3.

### PLA2G6 genotyping

To investigate how the two identified *PLA2G6* variants segregate in the family of the NAD-affected sheep, we genotyped all 71 available Swaledale sheep. The sire of the five cases was heterozygous for the exon 8 variant, and all the dams of the five NAD-affected lambs were heterozygous for the intron 2 variant (Fig. 2a). Interestingly, only the five affected animals carried both variants in a heterozygous state, which was consistent with a compound heterozygous inheritance (Fig. 2a). From the 13 clinically normal lambs, three were homozygous wild type for both *PLA2G6* variants, seven were heterozygous for the exon 8 variant and three were heterozygous for the intron 2 variant. In total, out of 52 genotyped ewes, 25 were homozygous wild type for both variants, and 27 were heterozygous for the intron 2 variant (Fig. 2a). Although, we had no sample of the reported common maternal grandsire, we concluded that either he was a carrier or a germline mosaic for the *PLA2G6* variant in intron 2.

### Functional confirmation of detected PLA2G6 variants

To assess the putative impact of the *PLA2G6* variant in intron 2 and the variant in exon 8, we experimentally analyzed both annotated *PLA2G6* transcripts using mRNA extracted from the nervous tissues of three NAD-affected lambs with the compound heterozygous genotypes. The presence of both *PLA2G6* variants in cDNA of three affected sheep was verified by Sanger sequencing after successful RT-PCR amplification. For the splice site variant at the end of intron 2 (c.210-2A > G) using primers located in the exon 2 and exon 4/5 boundary (Fig. 2c; Online Resource 5), a cDNA fragment of the expected size was obtained in all three studied tissues (spinal cord, sciatic nerve, and cerebellum). This single-nucleotide variant affects the canonical dinucleotide sequence for the U2-type GT-AG acceptor splice site at the end of intron 2 (Fig. 2b) and, therefore, was in silico predicted to disrupt splicing. Sanger sequencing of the RT-PCR amplicon confirmed the loss of the evolutionary strongly conserved splice site leading to a 10 bp deletion at the 5′-end of exon 3 by activation of a cryptic splice site (Fig. 2b). As a consequence of the mutant transcript, if translated, the PLA2G6 protein is predicted to be significantly truncated containing only 72 amino acids lacking more than 90% of the normal protein (p.Leu71TrpfsTer3; Fig. 2b). For the variant in *PLA2G6* exon 8 (c.1186C > T), primers located at the exons 6/7 and exons 12/13 boundaries were used and resulted in amplification of two cDNA fragments of the expected sizes in all three studied tissues (Fig. 2c; Online Resource 5). Sanger sequencing revealed that the obtained RT-PCR products correspond to the two annotated *PLA2G6* transcripts (XM_027968104.1 and XM_012175630.3; Fig. 2c, d). The identified exon 8 variant is predicted as a missense variant for the longer transcript (XM_012175630.3) leading to an exchange of leucine to phenylalanine in the longer protein isoform (XP_012031020.2: p.Leu396Phe; Fig. 2d). However, in the shorter transcript (XM_027968104.1), this variant is predicted as nonsense creating a premature stop codon (XP_027823905.1: p.Gln396*; Fig. 2d) that results in ~ 50% truncation of the resulting protein (Fig. 2e). Finally, both variants are assumed to severely affect the structure of PLA2G6 by truncating the functionally important domains (Fig. 2e).

## Discussion

As a result of characterization of the clinicopathological phenotype and evaluating the relatedness of the observed cases of lambs suspicious for an inherited neurodegenerative disorder, we were able to unravel the most likely genetic cause of a novel form of recessive NAD in Swaledale sheep. The two identified loss-of-function variants in intron 2 and exon 8 of *PLA2G6* are inherited in a compound heterozygous way. The intron 2 variant affects a highly conserved acceptor splice site leading to a premature stop codon. Similar splicing defect in the bovine *MFN2* gene is known to cause a recessively inherited form of degenerative axonopathy in cattle (Drögemüller et al. 2011). PLA2G6 function is important in regulation of physiological processes, such as calcium homeostasis, inflammation, and cell death (Ramanadham et al. 2015). More than half of the amino acid sequence builds protein interaction domains and motifs: the N-terminal domain, the ankyrin repeat domain, and the catalytic domain (Fig. 2e) (Ramanadham et al. 2015). The long isoform includes an additional 54-residue proline-rich sequence at position 396, where it substitutes glutamine in the short isoform. The long isoform is membrane-bound, while the short isoform is found in the cytoplasm (Malley et al. 2018). The herein presented evidence suggests that the affected lambs, which carry a copy of each mutant shorter transcript version of ovine *PLA2G6*, are not able to express this isoform of the encoded protein. We hypothesize that both identified loss-of-function variants in PLA2G6 transcripts are likely to be degraded by nonsense-mediated decay. Malik et al*.* (2008) described *Pla2g6*-null mice showing age-dependent neurologic impairment by 13 months of age. The neuropathological analysis of Swaledale sheep revealed axonal spheroids in the brain similar to those observed in mice (Malik et al. 2008) and human INAD (Gregory et al. 2008). Taken together, the two described pathogenic variants (*PLA2G6*: c.210-2A > G and c.1186C > T) are considered disease-causing for this novel form of ovine NAD.

## Conclusion

Based on the known function of *PLA2G6* and its role in human and dog neurodegenerative disease, the rarity of the two identified variants in sheep and the perfect co-segregation of the variant alleles with the disease phenotype in the studied pedigree, we conclude that inherited NAD in Swaledale sheep is caused by compound heterozygosity for the two identified loss-of-function variants in *PLA2G6*. Thereby, this study provides another example for the occurrence of allelic heterogeneity causing Mendelian disorders in a livestock species. Our study is the first report of the precise underlying pathogenesis of ovine NAD and demonstrates the success of the WGS-based precision medicine approach using the parent–offspring trios in detecting pathogenic variants associated with rare neurodegenerative disease.

## Electronic supplementary material

Below is the link to the electronic supplementary material.

Online Resource 1 (.mp4) Video illustrating the clinical phenotype of three NAD-affected Swaledale sheep. (MP4 92698 KB)

Online Resource 2 (.xlsx) NAD-linked genome regions with positive LOD scores in non-parametric linkage analysis. (XLSX 14 KB)

Online Resource 3 (.xlsx) List of whole-genome sheep sequences. (XLSX 14 KB)

Online Resource 4 (.xlsx) Private protein-changing variants observed in the sequenced Swaledale family. (XLSX 11 KB)

Online Resource 5 (.xlsx) List of primers used for RT-PCR and genotyping of the *PLA2G6* variants. (XLSX 11 KB)

## Data Availability

The whole-genome data of our group has been made freely available under study accession number PRJEB30931 in the European Nucleotide Archive (https://www.ebi.ac.uk/ena). All accession numbers of the WGS are available in the Online Resource 3. Sheep genomes project variant database of further 453 samples is deposited in ENA under accession number PRJEB14685. All references to the ovine *PLA2G6* gene correspond to the accessions NC_040254.1 (NCBI accession), XM_027968104.1 and XM_012175630.3 (mRNA), and XP_027823905.1 and XP_012031020.2 (protein).

## References

[CR1] Abecasis GR, Cherny SS, Cookson WO, Cardon LR (2002). Merlin—rapid analysis of dense genetic maps using sparse gene flow trees. Nat Genet.

[CR2] Baburina I, Jackowski S (1999). Cellular responses to excess phospholipid. J Biol Chem.

[CR3] Chang CC, Chow CC, Tellier LCAM (2015). Second-generation PLINK: rising to the challenge of larger and richer datasets. Gigascience.

[CR4] Cordy DR, Richards WPC, Bradford GE (1967). Systemic neuroaxonal dystrophy in suffolk sheep. Acta Neuropathol.

[CR5] Drögemüller C, Reichart U, Seuberlich T (2011). An unusual splice defect in the mitofusin 2 gene (MFN2) is associated with degenerative axonopathy in tyrolean grey cattle. PLoS ONE.

[CR6] Finnie JW, Jerrett IV, Manavis J, Cave J (2014). Neuroaxonal dystrophy in merino-border leicester × polled dorset lambs. Aust Vet J.

[CR7] Fyfe JC, Al-Tamimi RA, Liu J (2011). A novel mitofusin 2 mutation causes canine fetal-onset neuroaxonal dystrophy. Neurogenetics.

[CR8] Garcia JP, Giannitti F, Finnie JW (2015). Comparative neuropathology of ovine enterotoxemia produced by clostridium perfringens type D wild-type strain CN1020 and its genetically modified derivatives. Vet Pathol.

[CR9] Gregory A, Westaway SK, Holm IE (2008). Neurodegeneration associated with genetic defects in phospholipase A(2). Neurology.

[CR10] Hahn K, Rohdin C, Jagannathan V (2015). TECPR2 associated neuroaxonal dystrophy in Spanish water dogs. PLoS ONE.

[CR11] Hanshaw DM, Finnie JW, Manavis J, Kessell AE (2015). Axonal spheroid accumulation in the brainstem and spinal cord of a young angus cow with ataxia. Aust Vet J.

[CR12] Harper P, Morton A (1991). Neuroaxonal dystrophy in Merino sheep. Aust Vet J.

[CR13] Hawes MC, Finnie JW, Jerrett IV (2017). Primary, congenital neuroaxonal dystrophy with peripheral nerve demyelination in Merino-Border Leicester × Polled Dorset lambs. Aust Vet J.

[CR14] Hayflick SJ, Kurian MA, Hogarth P (2018). Neurodegeneration with brain iron accumulation. Handb Clin Neurol.

[CR15] Kessell AE, Finnie JW, Blumbergs PC (2012). Neuroaxonal dystrophy in Australian Merino lambs. J Comp Pathol.

[CR16] Lucot KL, Dickinson PJ, Finno CJ (2018). A missense mutation in the vacuolar protein sorting 11 (VPS11) gene is associated with neuroaxonal dystrophy in rottweiler dogs. G3 Genes Genom Genet.

[CR17] Malik I, Turk J, Mancuso DJ (2008). Disrupted membrane homeostasis and accumulation of ubiquitinated proteins in a mouse model of infantile neuroaxonal dystrophy caused by PLA2G6 mutations. Am J Pathol.

[CR18] Malley KR, Koroleva O, Miller I (2018). The structure of iPLA2β reveals dimeric active sites and suggests mechanisms of regulation and localization. Nat Commun.

[CR19] Naval-Sanchez M, Nguyen Q, McWilliam S (2018). Sheep genome functional annotation reveals proximal regulatory elements contributed to the evolution of modern breeds. Nat Commun.

[CR20] Nuttall WO (1988). Ovine neuroaxonal dystrophy in New Zealand. N Z Vet J.

[CR21] Paris JM, Letko A, Häfliger IM (2019). Identification of two TYRP1 loss-of-function alleles in Valais Red sheep. Anim Genet.

[CR22] Ramanadham S, Tomader A, Ashley JW (2015). Calcium-independent phospholipases A2 and their roles in biological processes and diseases. J Lipid Res.

[CR23] Sisó S, Hanzlíček D, Fluehmann G (2006). Neurodegenerative diseases in domestic animals: a comparative review. Vet J.

[CR24] Thorvaldsdóttir H, Robinson JT, Mesirov JP (2013). Integrative genomics viewer (IGV): high-performance genomics data visualization and exploration. Brief Bioinform.

[CR25] Tsuboi M, Watanabe M, Nibe K (2017). Identification of the PLA2G6 c.1579G>A missense mutation in papillon dog neuroaxonal dystrophy using whole exome sequencing analysis. PLoS ONE.

[CR26] Ye J, Coulouris G, Zaretskaya I (2012). Primer-BLAST: a tool to design target-specific primers for polymerase chain reaction. BMC Bioinform.

